# Achillea Millefolium L. Hydro- Alcoholic Extract Protects Pancreatic Cells by Down Regulating IL- 1β and iNOS Gene Expression in Diabetic Rats

**Published:** 2014

**Authors:** Yalda Zolghadri, Mehdi Fazeli, Marzieh Kooshki, Tahoora Shomali, Negar Karimaghayee, Maryam Dehghani

**Affiliations:** *Division of Pharmacology and Toxicology, Department of Basic Sciences, School of Veterinary Medicine, Shiraz University, Shiraz, Iran.*

**Keywords:** *Achillea millefolium L.*, IL-1β, iNOS, diabetes, gene expression

## Abstract

Interleukin-1β (IL-1β) has a role in β- cell destruction in autoimmune diabetes by stimulating the expression of inducible nitric oxide synthase (iNOS) that generates the free radical nitric oxide. We aimed to investigate the effect of *Achillea millefolium *L, as a traditional hypoglycemic agent, on IL-1β and iNOS gene expression of pancreatic tissue in the STZ- induced diabetic rats. Forty adult male Wistar rats were randomly divided into four groups: 1. diabetic control; 2. diabetic rats treated with *Achillea millefolium *L*.* extract; 3. normal rats received only extract and 4. negative control (n= 10 each). Diabetes was induced by single i.p. injection of 45 mg/ kg streptozotocin (STZ). Rats in groups 2 and 3 were treated with i.p. injection of *Achillea millefolium L.* extract (100 mg/ kg/ day) for 14 days. Body weight, serum glucose and insulin levels were assayed at baseline and on days 3, 7, 10 and 14 of the experiment. Finally, the quantity of pancreatic IL-1β and iNOS mRNA was determined by real- time PCR. The mRNA expression level of IL-1β and iNOS genes, was significantly (*p*<0.001) increased in diabetic rats of group 1. Treatment with *Achillea millefolium* L. caused a significant (*p*<0.01) reduction in both IL-1β and iNOS genes expression. Moreover, rats in group 2 had higher insulin level associated with lower glucose level and higher body weight compared to control diabetic group. It seems that beneficial effect of *Achillea millefolium *L. on STZ- induced diabetes is at least partly due to amelioration of IL-1β and iNOS gene over expression which can have a β-cell protective effect.

Type 1 diabetes mellitus (T1D) is an autoimmune disease which is associated with selective destruction of insulin producing β-cells (1). Both genetic and environmental factors play important roles in the etiology of the disease (2). Oxidative stress depicts the existence of products called free radicals and reactive oxygen species (ROS) which are formed under normal physiological conditions but become deleterious when not being quenched by the antioxidant systems (3). ROS generation is increased in both types of diabetes, showing the close association of diabetes and oxidative stress  (4, 5). Free radicals are formed disproportionately by glucose autoxida-tion, polyol pathway and non- enzymatic glycation of proteins in diabetes (6). Abnormal high levels of free radicals and simultaneous decline of antioxidant defense systems can lead to cellular damage, increased lipid peroxidation and development of diabetes mellitus complications (7).

Interleukin-1β (IL-1β) has been implicated as an effector molecule of β-cell destruction in autoimmune diabetes (8, 9). This cytokine is increased in patients with newly diagnosed T1D and likely acts as an early inflammatory signal in T1D development (10). It is possible that IL-1β acts in concert with other cytokines to cause islet cell death and it has been demonstrated that the cytotoxic effect of IL-1β is associated with the expression of inducible nitric oxide synthase (iNOS) and production of nitric oxide      (11).

Several *Achillea* species are used for their pharmaceutical, cosmetic and fragrance properties. Their extracts exhibit pharmacological activities such as anti-inﬂammatory, analgesic and antipyretic. *Achillea millefolium L.* (yarrow) has shown a wide range of therapeutic applications such as treating wounds, colds, fevers, kidney diseases, stopping blood ﬂow and reducing menstrual pain (12). It has traditionally been used as a hypoglycemic agent in diabetic status (-), however the mechanism(s) behind its hypoglycemic effect are not fully elucidated, thus this study aimed to investigate the effect of *Achillea millefolium L.* on IL-1β and iNOS gene expression of pancreatic tissue in the STZ- induced diabetic rats.

## Materials and Methods

**Plant material**

Aerial parts of the plant were collected from a local store and identiﬁed by the Department of Cultivation and Development, Institute of Medicinal Plants, Tehran, Iran. The dried and milled powdered plant material (120 g) was extracted with 1800 ml ethanol (80%) by percolation procedure in three steps then the ethanolic extract was dried by evaporation in temperature between 35- 40 °C (16 with slight modifications).

**Animals and experimental design**

Male Wistar albino rats (n= 40), 6- 8 months old, with a body weight of 200–250g were purchased from Razi institute, Shiraz, Iran. They were housed under conventional conditions and had free access to food and water. After acclimatization, animals were randomly divided into four groups: 1. diabetic control; 2. diabetic rats treated with *Achillea millefolium L.* extract; 3. normal rats received only extract and 4. negative control (no drug treatment at all) (n=10 in each group).

On day 0 of the experiment, diabetes was induced by intraperitoneal (i.p.) injection of streptozotocin (STZ; Sigma, USA; 45 mg/kg body weight). Streptozotocin was dissolved in 0.1 M sodium citrate buffer at pH 4.5 immediately before being injected (17). Three days after STZ administration, fasting blood glucose levels higher than 235 mg/ dl was considered as diabetic phenotype which was observed in all STZ- treated rats.

STZ- treated rats received 5% glucose in their drinking water for the first 24 h to counter any initial hypoglycemia. Animals in groups 3 and 4 were similarly injected with vehicle only.

Rats in groups 2 and 3 were treated with i.p. injection of *Achillea millefolium L.* extract (100 mg/ kg) for 14 days from day 0. The control healthy rats (negative control) and the control diabetic rats received the same volume of distilled water.

All procedures used in the present study are in accordance with ethical guidelines of School of Veterinary medicine, Shiraz University for care and use of laboratory animals in experimental studies.

**Measurement of biochemical parameters**

Body weight, serum glucose and insulin levels were assayed at baseline and on days 3, 7, 10 and 14 of the experiment. Blood samples were taken after an overnight fasting. Blood samples were centrifuged at 4 ^o^C for 15 min at 3000 rpm and the sera harvested were used for biochemical analyzes. Fasting blood glucose was assayed by commercially available glucose kit based on automated glucose oxidase method with the Glucose Analyser 2 (Beckman Coulter Inc., Fullerton, CA, USA) and the plasma insulin was measured using an ELISA kit (Linco Research Inc., MO, USA).

**IL-1β and iNOS gene expression analyses using real- time PCR**

Immediately after blood sampling, pancreas was quickly removed from the sacrificed rats under deep chloroform anaesthesia and was immediately frozen in liquid nitrogen and kept in -70 °C until use. The quantity of pancreatic IL-1β and iNOS mRNA was determined by quantitative real- time PCR. Pancreatic total RNA was extracted using RNX-Plus commercial kit (CinnaGen Inc. Tehran, Iran) according to the manufacturer’s instructions. After DNase (Fermentas, Vilnius, Lithuania) treatment and normalization by spectrophotometric method, 1 μg of RNA was reverse transcribed to ﬁrst- strand cDNA according to the standard protocol. The obtained cDNA was subjected to PCR amplification to estimate the expression of IL-1β and iNOS genes. A constitutive expression gene, the glyceraldehyde phosphate dehydrogenase (GAPDH), was used as internal control to verify the real- time PCR reaction. The primer sequences used were as follows: (1) IL-1β: forward primer: 5΄-CAGAATCTATACCTGTCCTG- 3΄, reverse primer: 5΄-TGCAGACTCAAACTCCACT- 3΄ yielding a 129 bp size product; (2) iNOS: forward primer: 5΄-GGTGTTCTTTGCTTCTGTGCTAAT- 3΄, reverse primer: 5΄-CGTGTTTGCCTTATACT-GTTCCA- 3΄; yielding a 157 bp size product and (3) GAPDH: forward primer: 5΄- 3΄, reverse primer: 5΄-GTACTCAGCACCAGCATCACC- 3΄ yielding a 153 bp size product. The PCR conditions were an initial denaturation for 5 min at 94 °C, 40 cycles with denaturation at 94 °C for 30 seconds, primer annealing at 51 °C (IL-1β), 54 °C (iNOS) and 57 °C (GAPDH) for 40 seconds and primer extension at 72 °C for 45 seconds and a final extension step at 72 °C for 5 min. The quantitative RT-PCR was performed by a real time PCR kit (Bioneer, Seoul, South Korea) using SYBR Green in a MiniOpticon^TM^ thermocycler (Bio-Rad laboratories Inc., CA, USA). DEPC- water for the replacement of cDNA template was used as negative control. The SYBR Green RT-PCR assay was carried out as previously described in detail (18). The results for IL-1β mRNA levels were presented relative to the expression of GAPDH (a housekeeping gene).

**Statistical analysis**

Data were presented as mean± SD. Data analysis was carried out by using one- way ANOVA and Tukey’s multiple comparison test as the *post hoc *(SPSS 11.5 for windows software). Differences were considered significant at *p*<0.05.

## Results

Diabetic rats in groups 1 and 2 had significantly lower body weight compared to negative control in days 7 and 10 post STZ injection (P<0.05).The administration of *Achillea millefolium *L. extract to STZ- diabetic rats restored the body weight to reach statistically the same value of negative control at day 14 (P>0.05). From day 10, administration of extract to diabetic rats of group 2 significantly reduced STZ- induced hyperglycaemia as compared to group 1 and on day 14 there was no significant difference in blood glucose level of rats in groups 2, 3 and 4. Serum insulin level of rats in group 2 was also reversed to negative control levels from day 10 post STZ injection. All measured parameters of rats in group 3 remained statistically the same as negative control during the experiment (Table 1). 

**Fig. 1:**
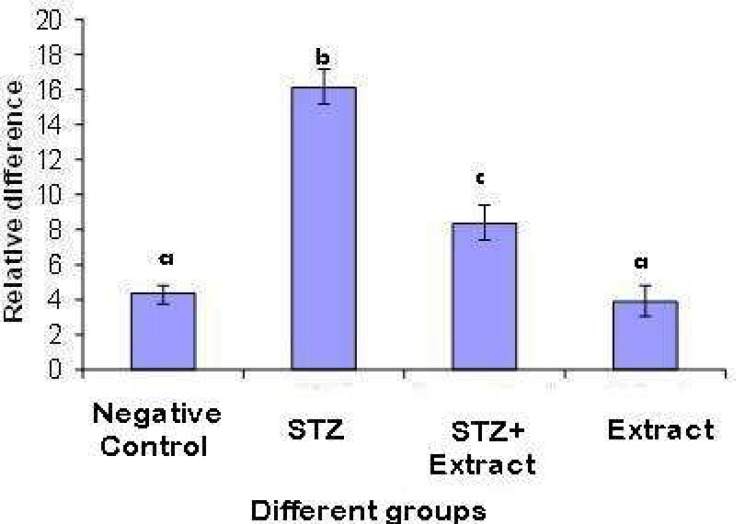
Relative difference (mean ± SD) in IL-1β expression in pancreatic tissues of rats in different groups. Different letters are used to demonstrate significant differences(p< 0.05). The IL-1β mRNA expression level was estimated using quantitative real-time RT-PCR and expressed relative to the expression level of GAPDH gene

**Fig. 2:**
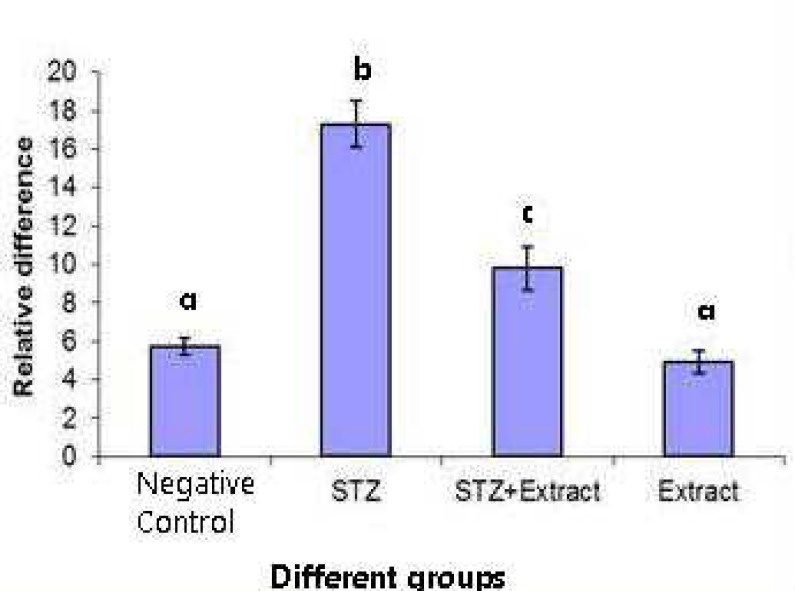
Relative difference (mean ± SD) in iNOS expression in pancreatic tissues of rats in different groups. Different letters are used to demonstrate significant differences (p< 0.05). The level of iNOS mRNA expression was estimated by quantitative real-time RT-PCR and normalized to the expression level of GAPDH gene

The mRNA expression level of IL-1β and iNOS genes assessed by real- time PCR, was significantly (P<0.001) increased in diabetic rats of group 1 as compared to negative control. Treatment with *Achillea millefolium* L. caused a significant (P<0.01) reduction in both IL-1β and iNOS genes expression (about 56 and 55 percent, respectively). The IL-1β/ GAPDH mRNA ratio was 3.76 fold higher in diabetic rats compared with that in control ones and treatment with extract reduced the ratio to 1.92 fold (Fig. 1). The ratio of iNOS/ GAPDH mRNA was 3 fold greater in diabetic rats as compared with that in control ones and was reduced to 1.76 fold after treatment with extract (Fig. 2).

**Table 1:** Body weight (g), serum glucose (mg/dl) and insulin level (µU/ml) (mean±SEM) of rats in different sampling times (n=10 in each group).

**Time (Days)** **after STZ injection**	**Parameters**	**Groups**			
		**1** **(Diabetic Control)**	**2** **(Diabetic+** **Extract)**	**3** **(Extract)**	**4** **(Negative Control)**
0	Body weight (g)	217 ± 3.67	221 ± 4.82	218 ± 5.40	216 ± 4.23
	Blood glucose (mg/dl)	97.33 ± 3.19	100.83 ± 4.59	97.67 ± 7.87	104.67 ± 4.42
	Serum insulin (µU/ml)	17.86 ± 1.06	16.32 ± 0.76	18.24 ± 0.89	17.65 ± 1.33
3	Body weight (g)	214 ± 4.21	212 ± 3.88	214 ± 5.12	221 ± 3.95
	Blood glucose (mg/dl)	332.83 ± 18.72^a^	345.00 ± 14.70^a^	101.50 ± 3.53^b^	103.00 ± 6.51^b^
	Serum insulin (µU/ml)	13.42 ± 0.87^a^	14.38 ± 0.69^a^	16.12 ± 1.04^b^	17.02 ± 0.96^b^
7	Body weight (g)	202 ± 4.96^a^	195 ± 4.22^a^	219 ± 5.01^b^	213 ± 3.89^b^
	Blood glucose (mg/dl)	380.67 ± 13.59^a^	370.83 ± 9.60^a^	109.33 ± 7.30^b^	110.33 ± 7.55^b^
	Serum insulin (µU/ml)	12.09 ± 0.66^a^	12.31 ± 0.33^a^	15.66 ± 1.13^b^	18.12 ± 1.05^b^
10	Body weight (g)	196 ± 4.21^a^	204 ± 3.97^a^	218 ± 4.32^b^	211 ± 5.23^b^
	Blood glucose (mg/dl)	392.00 ± 11.78^a^	304.17 ± 9.12^b^	102.67 ± 6.09^c^	105.17 ± 4.04^c^
	Serum insulin (µU/ml)	11.76 ± 0.47^a^	14.89 ± 0.95^b^	15.84 ± 0.90^b^	16.21 ± 1.18^b^
14	Body weight (g)	188 ± 4.71^a^	212 ± 3.27^b^	220 ± 4.03^b^	217 ± 3.77^b^
	Blood glucose (mg/dl)	403.50 ± 6.99^a^	187.17 ± 12.02^b^	107.17 ± 4.39^b^	107.50 ± 6.29^b^
	Serum insulin (µU/ml)	11.50 ± 0.27^a^	15.72 ± 0.56^b^	14.92 ± 1.00^b^	15.96 ± 0.92^b^

Different superscript small lettersis used to show significant differences in the same row (p<0.05).

Nevertheless, the administration of the extract could not completely reverse increased expression of both genes to negative control levels. The expression of neither genes did show any considerable changes in the normal rats treated with *Achillea millefolium* L*.* extract (Fig.1 and 2).

## Discussion

Traditional plant treatments have been used all over the world for diabetes (19). In recent years, dietary plants with antioxidant property have gained lots of credits from researchers in related fields and investigations on plants with hypoglycaemic and antioxidative chracteristics have grown rapidly. It is believed that these plants can protect tissues against deleterious effects of free radicals which can lead to cytokine activation (20). *Achillea millefolium* L. is among the medicinal herbs with antioxidant and hypoglycaemic properties. It has been shown that *Achillea millefolium *L. essential oils inhibit LPS- induced oxidative stress and nitric oxide production in mouse leukaemic monocyte macrophage cell line (RAW 264.7) (21). Moreover;* Achillea millefolium* L. extract has decreased blood glucose level and prevented the β-cells of pancreas from the cytotoxic effects of alloxan (22).

T1D is believed to be an immune- mediated process in which pro- inflammatory cytokines particularly IL- 1β plays an essential role (23, 24). Furthermore, the role of IL- 1β in T2D has also been established (25, 26). IL-1 β has been implicated in early events in β-cell destruction. Suppression of IL- 1β production or inhibition of its interaction with corresponding cellular receptors significantly inhibits IL- 1β-mediated deleterious effects on β–cells (27, 28) and direct blockade of IL-1β has been studied as a therapeutic strategy for T1D at the preclinical 

level (10). *In vitro* observations indicate that the cytotoxic effect of IL- 1β in islet cells involves the induction of nitric oxide synthase (iNOS) and the production of nitric oxide (29, 30).

STZ is commonly used to induce diabetes in the experimental animals (16, 31, 32). It destroys islet cells through several mechanisms, including production of reactive oxygen species (ROS) (33), activation of pancreatic NF-κβ (34) and induction of pronounced immune and inflammatory responses (35). It has been suggested that overproduction of free radical NO under the inﬂuence of STZ may play a crucial role in destruction of the β-cells during the development of T1D (36).

Taken all together, the present study assessed the effect of *Achillea millefolium* L. hydro-alcoholic extract on expression of IL -1β and iNOS genes in pancreatic tissues of STZ- induced diabetic rats which may help in better elucidation of possible mechanisms behind the beneficial effect of this plant on diabetes. We observed that the elevated level of both IL-1β and iNOS gene expression declined appreciably due to administration of *Achillea millefolium *L. extract although these parameters did not completely reversed to normal values as compared with negative control rats.

IL -1β exerts its main effects through the NF-κβ pathway (37, 38). As previously mentioned in our study, *Achilea millefolium* L. extract caused a significant decrease in IL -1β and iNOS genes mRNA expression, which can combat the cytotoxic effect of STZ on pancreatic β-cells that was reflected in higher insulin level associated with lower glucose level and higher body weight of diabetic rats that received the extract compared to control diabetic group. It is anticipated that this beneficial effect might be mediated, at least in part, by inhibitory effect of antioxidants presented in flavonoid contents of the extract on NF-κβ activation. Flavonoids comprise the most common group of plant polyphenols and provide much of the antioxidative activity of fruits and vegetables. Several flavonoids have been shown to inhibit the expression of NF-κB- dependent cytokines, iNOS, and cyclooxygenase-2 genes (39). This needs to be further evaluated in future studies.

In conclusion it seems that beneficial effect of *Achillea millefolium* L. on STZ-induced diabetes is at least partly due to amelioration of IL-1β and iNOS gene over expression which can have a β-cell protective effect.

## References

[1] Atkinson MA, Eisenbarth GS (2001). Type 1 diabetes: new perspectives on disease pathogenesis and treatment. Lancet.

[2] Kuzuya T, Nakagawa S, Satoh J (2002). Report of the Committee on the classification and diagnostic criteria of diabetes mellitus. Diabetes Res Clin Pract.

[3] Fang YZ, Yang S, Wu G (2002). Free radicals, antioxidants, and nutrition. Nutrition.

[4] Johansen JS, Harris AK, Rychly DJ (2005). Oxidative stress and the use of antioxidants in diabetes: linking basic science to clinical practice. Cardiovasc Diabetol.

[5] Rosen P, Nawroth PP, King G (2001). The role of oxidative stress in the onset and progression of diabetes and its complications: a summary of a Congress Series sponsored by UNESCO-MCBN, the American Diabetes Association and the German Diabetes Society. Diabetes Metab Res Rev.

[6] Wolff SP, Dean RT (1987). Glucose autoxidation and protein modification The potential role of 'autoxidative glycosylation' in diabetes.. Biochem J.

[7] Maritim AC, Sanders RA, Watkins JB (2003). 3rd Diabetes, oxidative stress, and antioxidants: a review. J Biochem Mol Toxicol.

[8] Rabinovitch A, Suarez- Pinzon WL (1998). Cytokines and their roles in pancreatic islet beta-cell destruction and insulin-dependent diabetes mellitus. Biochem Pharmacol.

[9] Song MY, Bae UJ, Lee BH (2010). Nardostachys jatamansi extract protects against cytokine-induced beta-cell damage and streptozotocin- induced diabetes. World J Gastroenterol.

[10] Grishman EK, White PC, Savani RC (2012). Toll-like receptors, the NLRP3 inflammasome, and interleukin-1beta in the development and progression of type 1 diabetes. Pediatr Res.

[11] Holohan C, Szegezdi E, Ritter T (2008). Cytokine- induced beta-cell apoptosis is NO-dependent, mitochondria-mediated and inhibited by BCL-XL. J Cell Mol Med.

[12] Conforti F, Loizzo MR, Statti GA (2005). Comparative radical scavenging and antidiabetic activities of methanolic extract and fractions from Achillea ligustica ALL. Biol Pharm Bull.

[13] Petlevski R, Hadzija M, Slijepcevic M (2001). Effect of 'antidiabetis' herbal preparation on serum glucose and fructosamine in NOD mice. J Ethnopharmacol.

[14] McCune LM, Johns T (2002). Antioxidant activity in medicinal plants associated with the symptoms of diabetes mellitus used by the indigenous peoples of the North American boreal forest. J Ethnopharmacol.

[15] Jarald E, Joshi SB, Jain DC (2008). Diabetes Vs herbal medicines. Iran J Pharmacol Ther.

[16] Nilforoushzadeh MA, Shirani-Bidabadi L, Zolfaghari-Baghbaderani A (2008). Comparison of Thymus vulgaris (Thyme), Achillea millefolium (Yarrow) and propolis hydroalcoholic extracts versus systemic glucantime in the treatment of cutaneous leishmaniasis in balb/c mice. J Vector Borne Dis.

[17] Gomathi D, Ravikumar G, Kalaiselvi M (2013). Efficacy of Evolvulus alsinoides (L ) L. on insulin and antioxidants activity in pancreas of streptozotocin induced diabetic rats.. J Diabetes Metab Disord.

[18] Muller PY, Janovjak H, Miserez AR (2002). Processing of gene expression data generated by quantitative real-time RT-PCR. Biotechniques.

[19] Bailey CJ, Day C (1989). Traditional plant medicines as treatments for diabetes. Diabetes Care.

[20] Osawa T, Kato Y (2005). Protective role of antioxidative food factors in oxidative stress caused by hyperglycemia. Ann N Y Acad Sci.

[21] Chou S-T, Peng H-Y, Hsu J-C (2013). Achillea millefolium L. essential oil inhibits LPS-induced oxidative stress and nitric oxide production in RAW 264.7 macrophages. Int J Mol Sci.

[22] Mustafa KG, Ganai BA, Akbar S (2012). β-Cell protective efficacy, hypoglycemic and hypolipidemic effects of extracts of Achillea millifolium in diabetic rats. Chinese Journal of Natural Medicines.

[23] Sathyapalan T, Atkin S (2011). Is there a role for immune and anti-in-flammatory therapy in type 2 diabetes?. Minerva endocrinologica.

[24] Eizirik DL, Mandrup-Poulsen T (2001). A choice of death--the signal- transduction of immune-mediated beta- cell apoptosis. Diabetologia.

[25] Feve B, Bastard JP (2009). The role of interleukins in insulin resistance and type 2 diabetes mellitus. Nat Rev Endocrinol.

[26] Maedler K, Sergeev P, Ris F (2002). Glucose-induced beta cell production of IL-1beta contributes to glucotoxicity in human pancreatic islets. J Clin Invest.

[27] Tellez N, Montolio M, Estil-les E (2007). Adenoviral overproduction of interleukin-1 receptor antagonist increases beta cell replication and mass in syngeneically transplanted islets, and improves metabolic outcome. Diabetologia.

[28] Giannoukakis N, Rudert WA, Ghivizzani SC (1999). Adenoviral gene transfer of the interleukin-1 receptor antagonist protein to human islets prevents IL-1beta-induced beta-cell impairment and activation of islet cell apoptosis in vitro. Diabetes.

[29] Hoorens A, Stange G, Pavlovic D (2001). Distinction between interleukin-1-induced necrosis and apoptosis of islet cells. Diabetes.

[30] Steer SA, Scarim AL, Chambers KT (2006). Interleukin-1 stimulates beta-cell necrosis and release of the immunological adjuvant HMGB1. PLoS Med.

[31] Szkudelski T (2001). The mechanism of alloxan and streptozotocin action in B cells of the rat pancreas. Physiol Res.

[32] Murali B, Goyal RK (2002). Effect of chronic treatment with losartan on streptozotocin induced diabetic rats. Indian J Exp Biol.

[33] Schiekofer S, Andrassy M, Chen J (2003). Acute hyperglycemia causes intracellular formation of CML and activation of ras, p42/44 MAPK, and nuclear factor kappaB in PBMCs. Diabetes.

[34] Weiss RB (1982). Streptozocin: a review of its pharmacology, efficacy, and toxicity. Cancer Treat Rep.

[35] Karunanayake EH, Baker JR, Christian RA (1976). Autoradiographic study of the distribution and cellular uptake of (14C) - streptozotocin in the rat. Diabetologia.

[36] Haluzik M, Nedvidkova J (2000). The role of nitric oxide in the development of streptozotocin-induced diabetes mellitus: experimental and clinical implications. Physiol Res.

[37] Arnush M, Heitmeier MR, Scarim AL (1998). IL-1 produced and released endogenously within human islets inhibits beta cell function. J Clin Invest.

[38] Corbett JA, McDaniel ML (1995). Intraislet release of interleukin 1 inhibits beta cell function by inducing beta cell expression of inducible nitric oxide synthase. J Exp Med.

[39] Moussaieff A, Shohami E, Kashman Y (2007). Incensole acetate, a novel anti-inflammatory compound isolated from Boswellia resin, inhibits nuclear factor-kappa B activation. Mol Pharmacol.

